# Raman Spectroscopy and Machine Learning for Agricultural Applications: Chemometric Assessment of Spectroscopic Signatures of Plants as the Essential Step Toward Digital Farming

**DOI:** 10.3389/fpls.2022.887511

**Published:** 2022-04-26

**Authors:** Charles Farber, Dmitry Kurouski

**Affiliations:** ^1^Department of Biochemistry and Biophysics, Texas A&M University, College Station, TX, United States; ^2^Department of Biomedical Engineering, Texas A&M University, College Station, TX, United States; ^3^Department of Molecular and Environmental Plant Science, Texas A&M University, College Station, TX, United States

**Keywords:** chemometrics, Raman spectroscopy, plants, statistics, plant diseases

## Abstract

A growing body of evidence suggests that Raman spectroscopy (RS) can be used for diagnostics of plant biotic and abiotic stresses. RS can be also utilized for identification of plant species and their varieties, as well as assessment of the nutritional content and commercial values of seeds. The power of RS in such cases to a large extent depends on chemometric analyses of spectra. In this work, we critically discuss three major approaches that can be used for advanced analyses of spectroscopic data: summary statistics, statistical testing and chemometric classification. On the example of Raman spectra collected from roses, we demonstrate the outcomes and the potential of all three types of spectral analyses. We anticipate that our findings will help to design the most optimal spectral processing and preprocessing that is required to achieved the desired results. We also expect that reported collection of results will be useful to all researchers who work on spectroscopic analyses of plant specimens.

## Introduction

Predictive modeling suggests that the world’s population will be 9.8 billion people by the year 2050. In order to feed the world, we will need to produce about 70% more food than we do now (Payne and Kurouski, 2021a). This problem does not stand alone: If agricultural land expansion continues at its current rate, a 593 mega-hectare area approximately the size of India will be committed to agriculture (Searchinger et al., 2019). Such expansion would be fueled by the destruction of natural ecosystems around the world. There are many strategies to increase food yield without developing more agricultural land. Of these, protecting and improving existing crops are among the most important.

Plants constantly interact with the world around them (Fujita et al., 2006). When conditions are ideal, they can commit their energy stores to food production. However, plants can be affected by stresses based on interactions between themselves and other living organisms (biotic) or their environment (abiotic). Rapid detection, identification, and treatment of both biotic and abiotic stresses are key to improving crop yield around the world.

Biotic stresses are organisms that harm plants, including agricultural pests and pathogens. These stresses cause tremendous losses in many staple crops. Within the five most grown species (wheat, rice, maize, potato, and soybean), about 20–30% of the crop is lost each year due to pests and pathogens, with greater losses observed in regions with existing food security issues (Savary et al., 2019). For example, in the 2018/2019 growing year, an estimated 497 million metric tons (MMT) of rice was produced (Payne and Kurouski, 2021b). Without losses from pests and pathogens, between 596.4 and 646.1 MMT could have been produced. Control of pests and pathogens is essential to improving yield without expanding agricultural space.

In contrast, abiotic stresses are environmental factors that can harm plants. Example stresses include salt, water, temperature, nutrient deficiency, and misapplication of herbicides (Henry et al., 2004; Panta et al., 2014; Waqas et al., 2017). Abiotic stresses are constant issues in agriculture: Altogether, they are thought to be associated with 50–70% of all crop losses around the world (Dos Reis et al., 2012). The issues of abiotic stresses can be further complicated by their visual symptomatic similarity to biotic stresses, which can lead to wasted resources diagnosing for a pathogen instead of inadequate environmental conditions. Rapid response and treatment of abiotic stresses are critical to increase crop yield.

An equally important method for combatting plant stress is development of varieties with higher yield and tolerance to stresses. Despite great progress in genotyping technology, phenotyping has not progressed at the same rate (Forster et al., 2015). Most traits require that the plant be germinated and tested for a response. While these tests are generally quite simple, such as subjecting the plants to elevated salt levels to test for salt stress resistance, slow growth rates limit the scalability of this technique. A method that could correlate information about seeds or seedlings with their valuable traits would be powerful to accelerate plant breeding efforts.

To address these issues in agriculture, modern spectroscopic technologies that enable detection and identification biotic stresses, abiotic stresses, and plant varieties are required. Spectroscopic methods are based on various interactions between light and matter, and how these interactions change under stress or between varieties. Depending on the method, spectroscopy can provide rich chemical information about the sample, allowing the experimenter to connect spectral and chemical changes in the plant. These methods are typically fast but cannot survey entire fields at the rate of the imaging methods. Most spectroscopies are noninvasive and nondestructive, enabling their use in the field.

Vibrational spectroscopy methods use light to probe the vibrations of molecular bonds in the sample. Because each molecule has a unique set of bonds, these methods enable identification of chemicals in a sample by shining light on them alone, suggesting that these methods can be noninvasive and nondestructive. In plant science, vibrational spectroscopy can reveal the chemical composition differences between plants with different stresses or genetic backgrounds. In this thesis, two methods will be discussed: infrared in this section and Raman later. Infrared (IR) spectroscopy uses absorption of light in the middle-infrared range (2,500–20,000 nm) to identify the molecular bonds present in a sample. The specifics of an IR experiment depend on the instrument used, but the interpretation of the data remains uniform. For example, in a transmission infrared instrument, light is passed through the sample to a detector. In this case, light is absorbed as it passes through the sample. In an attenuated total reflection (ATR) instrument, in contrast, light is shined onto the sample surface at a specific angle that allows light absorption at a sample–crystal interface. The remaining light then travels to the detector. In either case, wavelengths absorbed by the sample will not reach the detector as much as those not absorbed. These variations in absorption/transmission can be plotted against excitation wavelength to create an infrared spectrum (Farber et al., 2019b). The greatest advantage of IR is its ability to associate chemical changes with differences between stresses or varieties of plants or pathogens. Abu-Aqil and colleagues illustrated this in their differentiation of phytopathogenic bacteria from two different genera and several strains (Abu-Aqil et al., 2020). Bacterial samples were isolated from infected plant material, cultured, then sampled using FT-IR following a brief drying period. The spectra largely showed the same bands across strains and species, but the intensities of the bands varied by genotype. Using principal component analysis and support vector machines, the team was able to discriminate between the different species and strains with high accuracy. The team demonstrated the great strength of IR: ability to identify the chemical makeup of the sample to further understand how the classification algorithms differentiate between the strains and genera. This study also identifies one of the limitations of IR spectroscopy—water. Water has a large, broad absorption band across the 0–1,000 cm^−1^ region of the spectrum which can obscure valuable sample information if not removed (Benjamin, 1994). This water issue also limits maximum distance between the sample and the IR detector, as water vapor in that space may interfere with the spectrum. A spectroscopic method insensitive to water and with the potential to generate spectra from great distances may be a useful complement for IR spectroscopy.

Raman spectroscopy (RS) is a form of vibrational spectroscopy, a family of methods that uses light to probe the vibrations of bonds within molecules. Vibrational methods enable the identification of molecules present in a sample. With this useful property, Raman has been described for use in a wide variety of fields, including forensics, polymer chemistry, and geology (Lippert et al., 1993; Sharma et al., 2003; Westley et al., 2017). Initially described by C.V. Raman and K.S. Krishnan in 1928, the Raman effect arises from interactions between matter and light (Raman and Krishnan, 1928). Raman spectroscopy is based on inelastic light scattering from a sample. Scattering causes the light to change directions and potentially energy level. Most scattering events are elastic (known as Rayleigh scattering), resulting in no energy change. These photons are excluded using optical filters. In an inelastic scattering event, photons exchange energy with molecules in the sample. In Stokes scattering, the photons lose energy, whereas in anti-Stokes, they gain energy. The ratio of Stokes/anti-Stokes scattering is dependent on the temperature of the analyzed system, but Stokes is more common than anti-Stokes at room temperature (25°C; Richard-Lacroix and Deckert, 2020). In a typical regular Raman experiment, the Stokes scattered light is analyzed rather than the anti-Stokes.

Both Stokes and anti-Stokes scattering can be amplified by localized surface plasmon resonances (LSRPs) that are generated on the surfaces of noble metal nanostructures upon their illumination by light. LSPR-enhanced Raman, also known as surface-enhanced Raman scattering (SERS) was first observed by Fleishman (Fleischmann et al., 1874) and the later explained by Van Duyne (Jeanmaire and Van Duyne, 1977). A growing body of evidence shows that SERS can be used to detect fungal toxins present at low concentrations in grain. Specifically, Lee et al. utilized SERS to quantify afatoxin, a metabolite produced by *Aspergillus favus*, in corn at a concentration range of 0–1,206 μg/kg (Lee et al., 2014). Using SERS, Kadam and co-workers were able to detect the transgene DNA from multiple transgenic lines of *Arabidopsis* (Kadam et al., 2017a). The authors demonstrated that SERS enabled quantification of the transgenes as low as 0.10 pg. without need for PCR amplification. The lrudayaraj group also showed that SERS could be used for detection and quantification of alternative splice sites in *Arabidopsis* genes AtDCL2 and AtPTB2 (Kadam et al., 2014b), as well as rare RNA transcripts from protoplasts in this plant species (Kadam et al., 2014a, 2017b). These examples demonstrate that SERS can be a good alternative to RS if additional sensitivity is required for diagnostics of the pathogens.

For this work, we collected Raman spectra from healthy rose plants, as well as plants diagnosed by rose rosette disease (diagnosed by PCR). In one of the infected group of plants, disease symptoms were visible (symptomatic group), whereas in another one symptoms were not visible (asymptomatic group).

## Materials and Methods

Leaf samples of The Double Knock Out^®^ Roses that were grown outdoors in College Station, Ferris, and Van Alstyne, TX, and Durant, OK, were collected and analyzed by both Raman and PCR testing (Farber et al., 2019a). Growing conditions were identical at all locations. Healthy plants were negative by PCR. All positive by PCR plants were divided into two group: symptomatic and asymptomatic based on the evidence of at least several rose rosette present on those plants (Farber et al., 2019a).

### PCR Analysis

We have used PCR testing procedures previously described by Farber and co-workers (Farber et al., 2019a). Briefly, plant leaves were homogenized in 1X phosphate-buffered saline that contained 0.05% Tween-20 (1X PBS-T). After the short incubation in 1X PBS-T, the homogenate was removed and the sample tube was washed twice with 50 μl 1X PBS-T. Next, the extracted RNA was resuspended in water and stabilized with RNase inhibitor (#N2511, Promega, Madison, WI). Virus diagnostics was performed using a One-Step RT-PCR kit (#210212, Qiagen, Valencia, CA) with the following primers: The primary uses to confirm the presence of the virus are as follows: RRVF (5′-GCACATCCAACACTCTTGCAGC-3′) and RRVR (5′-CTTATTTGAAGCTGCTCCTTGATTTCC-3′). PCR products were visualized by electrophoresis through 2% agarose gels for 1 h at 5v/cm in 1X TAE buffer (40 mM Tris, 20 mM Acetate, 1 mM EDTA, pH 8.6). The sizes of amplified DNA products were determined by using 50–2000 bp DNA Markers (HyperLadder^™^ 50 bp, Bioline, Memphis, TN). Expected product size is 271 bp. Presence/absence is determined based on bands, with faint bands being considered as inconclusive (Farber et al., 2019a).

### Raman Spectroscopy

Raman spectra were collected form fresh plant leaves using Agilent Resolve spectrometer. Laser power of 495 mW and 1 s acquisition time were used. Approximately 2–4 spectra were collected form one leaf. Spectra were acquired from leaf areas in-between the leaf veins. This approach allows for minimization of the spectral divergence caused by histological heterogeneity of plant leaves. Spectra were baselined automatically by the instrument software and imported into the MATLAB addon PLS_Toolbox 8.6.2 for statistical analyses (Farber et al., 2019a). We avoided to perform spectral analysis of leaves that had visual damages or yellowness. Typically, such leaves provided poor signal to noise spectra. All healthy-looking leaves exhibited Raman spectra with excellent signal to noise ratio.

## Results and Discussion

Collection of individual spectra is insufficient to make a conclusion about presence or absence of a biotic or an abiotic stress (Payne and Kurouski, 2021a). Plant tissues exhibit substantial anatomic and histological heterogeneity that will result in spectral variations. It is important to exclude the contribution of such heterogeneities to the reported spectra. Therefore, several rather than one spectrum is typically collected. In this case, statistical analysis of the spectra is required. There are three different levels of statistical analysis of spectra that will be discussed in this work: summary statistics, statistical testing, and chemometric classification. We will demonstrate the outcomes of each of these methods and will demonstrate advantages and disadvantages of each of these statistical approaches.

### Summary Statistics

Summary statistics are numbers that describe the trends of a dataset. These include measures of the following: central tendency such as the mean or median; dispersion such as the standard deviation or variance; shape such as the skew; and dependence such as correlation coefficients. For the purposes of Raman spectra, we are most often concerned with the central tendency, summarized with the mean, and the dispersion, summarized with the standard deviation.

**Figure 1:**
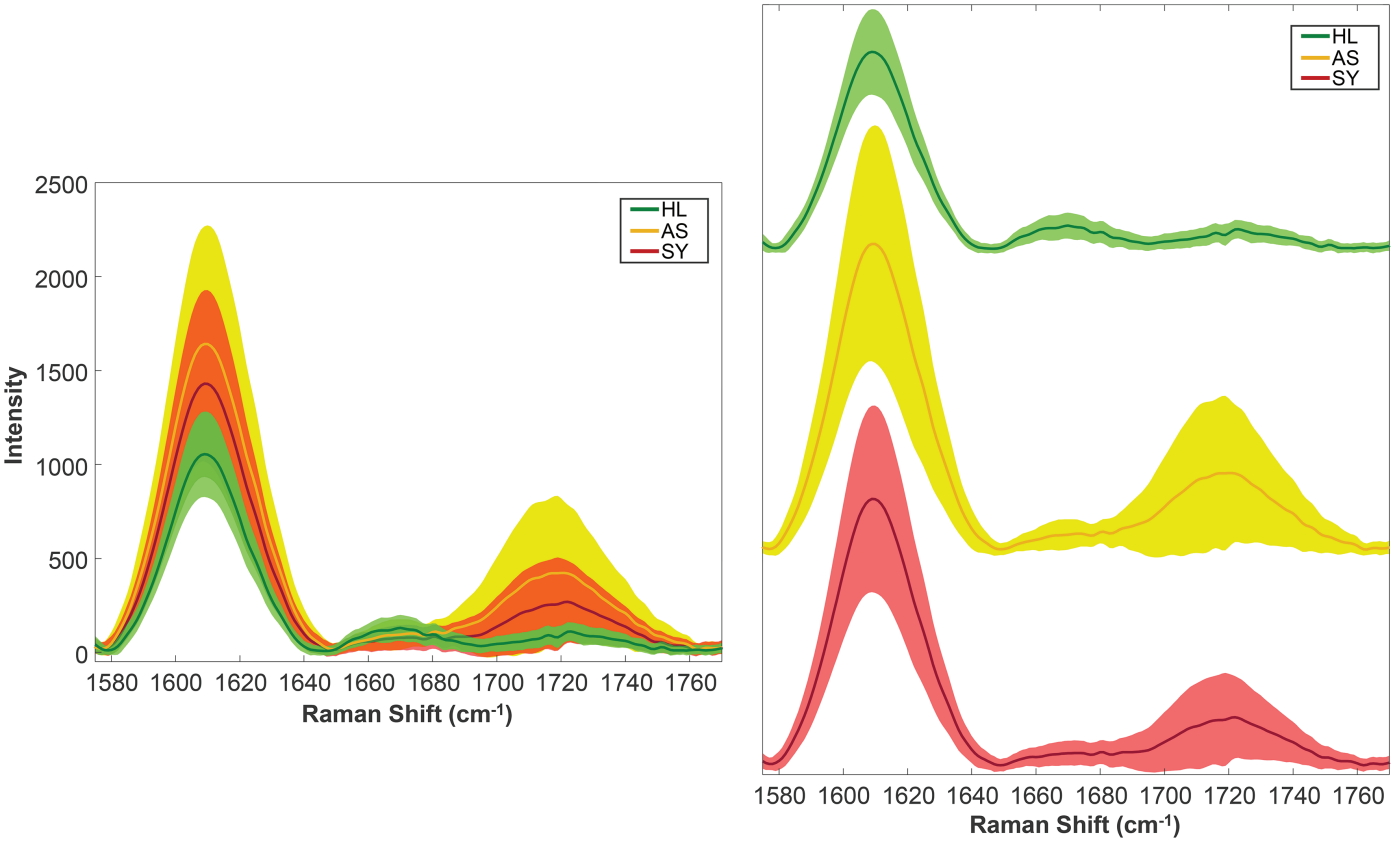
Rose leaf mean spectra (solid lines) and standard deviations (shaded regions). Left—stacked; right—offset vertically for clarity. HL—Healthy control; AS—asymptomatic; SY—symptomatic.

Figure 1 shows examples of mean spectra. Each of the three groups contains many spectra but averaging them together summarizes the data but may obscure spectral heterogeneity. In this case, the healthy group contains 134 spectra, the symptomatic group, 114 spectra, and the asymptomatic group, 441 spectra. On the right-hand side of the spectra, there appears to be differences in the intensities of the 1,610 and 1,720 cm^−1^ bands across the three groups. The mean, of course, does not tell the whole story.

By plotting the standard deviations, we can now observe how much each data group varies (Figure 1). As one might expect, the healthy group showed the least variability. Interestingly, the asymptomatic group showed the most variability. This suggests that the asymptomatic state represents a wide range of plants—from those just recently infected (potentially resembling healthy spectroscopically) to actively producing compounds to fight the infection, indicated by the elevated 1,610 cm^−1^ (aromatics) and 1,720 cm^−1^ (carbonyl) bands.

From these two summary statistics, we can make two general assertions: First, we have an idea of what the typical spectrum looks like, based on the mean. Second, we understand just how much our sample of spectra vary compared to the typical spectrum. Additionally, the control spectra were more uniform than the other groups, while the asymptomatic were the most variable.

There are many potential explanations as to why the spectra vary at all. In the context of these rose leaf spectra, it is possible that the assigned labels truly represent a wide range of conditions. For example, asymptomatic may include plants that are toward the beginning or end of the pathogen’s dormancy and are responding at different levels. On a general scale, the spectra could vary for many acquisition-related factors, such as presence of leaf veins or other variations in the leaf surface.

From summary statistics alone, we cannot make any further inferences such as: what is the chance to observe a control spectrum with intensity X at 1,610 cm^−1^ if the true mean intensity of the control spectra at that position is Y? For this, we need statistical testing.

### Statistical Testing

Statistical testing is a process by which a hypothesis about some trend in data can be evaluated. Using a statistical test, we can find the probability that we observe spectra like those in our dataset if our hypothesis is true. Each test starts with some hypothesis. For example, we may be interested in whether the mean intensities of the three groups at the 1,610 and 1,720 cm^−1^ bands are the same or not. In this case, the null hypothesis is: “The true mean intensities of the 1,610 cm^−1^ band in each group are the same.” We can summarize this as follows: μ_HL_ = μ_AS_ = μ_SY_, where μ_X_ is the population mean for group X. To be able to test this hypothesis, we must also outline the possible alternative. In this case, that alternative is “The true mean intensities at 1,610 cm^−1^ bands in at least two groups are not equal.” If our test concludes that there is a significant chance that any of μ_HL_, μ_AS_, or μ_SY_ are not equal, we have against the null hypothesis and in favor of the alternative. We would also construct the same hypothesis pairs for 1,720 cm^−1^ but conduct the tests separately.

The test we will conduct to test our hypothesis is called the ANOVA. ANOVA tests the question we set forth in our hypothesis: whether the means of the groups are different from each other or not. In some ways, this test is like a *t*-test, which is for comparison of two groups. ANOVA can compare many groups at once, whereas to compare all groups by t-testing, three separate tests are needed: HL v. AS; HL v. SY; and SY v. AS.

To conduct an ANOVA, the sample dataset must first meet a set of assumptions: The data must follow a normal distribution; the samples must be independent of each other; and the samples must have the same variance; and the data must come from a random sample. For independence and random sampling, based on the experimental design, we assume that the intensity of one spectrum does not depend on the intensity of the others and that the leaves were sampled randomly from the plants. For the other two assumptions, specific tests exist. Homogeneity of variance can be checked using procedures such as the Levene’s test (Levene, 1960). Normality can be tested with Anderson–Darling test or other methods (Anderson and Darling, 1954). From the Levene’s test, we found that our rose data does not meet the assumption of homogeneity of variance at either Raman shift. With the Anderson–Darling test, we found that only the healthy data at 1,720 cm^−1^ was normally distributed. Based on these results, we will need to use a different type of test.

Nonparametric statistical tests are methods which do not require an underlying distribution (i.e., normal distribution) to draw inferences from a dataset. The Kruskal–Wallis (KW) test is the nonparametric equivalent of the ANOVA (Kruskal and Wallis, 1952). The assumptions of the test are very simple: Data come from random sampling; the observations are independent; and the data can put in order (are ordinal), if they are not innately numerical. The rose data meets these assumptions, so we can proceed with the test.

With the nonparametric test, the procedure and outcome change slightly. First, instead of evaluating the intensity, we instead investigate the “rank” of the intensity of each spectrum. The spectral intensities at the band of interest, regardless of group membership, are sorted from smallest to largest and assigned a rank based on their position, with 1 being the smallest observation. Instead of evaluating the sample means, the sample median is evaluated instead. Our hypotheses change to “The medians of all groups are equal” and “The median of at least one group is not equal” for the null and alternative hypotheses, respectively. The results of our KW test were a Chi-square statistic of 70.08 and value of *p* = 6×10^−16^. This small value of *p* indicates that there is an extremely small chance that the medians of these groups are equal given the sample data. This suggests that we have found evidence to reject the null hypothesis in favor of the alternative, that at least one of the groups has a different median from the others.

**Figure 2:**
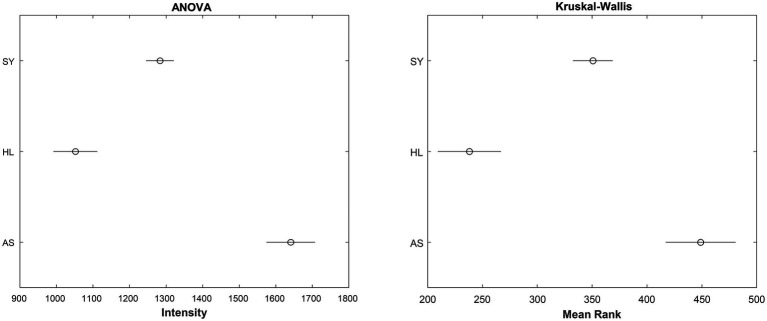
95% Confidence intervals for the true mean intensity (left) or mean rank of intensity (right) for the rose leaf data at the 1,610 cm^−1^ band. SY - symptomatic; HL - healthy control; AS - asymptomatic.

By itself, the KW test only indicates that a difference exists. It does not tell us which group is ifferent from the others. To identify this, a *post-hoc* test is needed. For the KW, the Dunn–Sidak test is considered the most appropriate (Šidák, 1967). Using this *post-hoc* test, we can obtain 95% confidence intervals for the mean ranks of the data. For the sake of comparison, the results of the ANOVA (though improper to conduct in this case) are plotted as well (Figure 2). Interestingly, despite failing the assumptions of the ANOVA, the outcomes of both tests are the same: All three groups are significantly different from each other at 1,610 cm^−1^.

The choice between ANOVA and alternative tests comes down to whether the data can meet the assumptions of ANOVA or not. One way to compare these two tests is by evaluating their power, or probability that the test rejects the null hypothesis when it is false. In their work, Hecke found that for a large, simulated dataset, when the ANOVA assumptions were met, the two tests had similar powers. However, when the assumption of normally distributed data was broken, KW had greater power (Hecke, 2012). This does not mean that KW should be used at all times, even when ANOVA assumptions are met. When using the nonparametric test such as KW, intensity information is replaced by ranks. While the ANOVA can help draw conclusions about the relationships between intensities across groups, KW can only provide the order of the groups because the analysis is based only on ranks.

From this result, we can say that the intensity ranks of the three treatments are significantly different from each other and infer on the biochemical changes that may result in this being the case. As shown in this section, statistical testing is not without great limitations, particularly if one wishes to use a parametric test. Additionally, these tests are limited to evaluating at single Raman shifts or specific areas under the curve. Because Raman spectra are relatively large (1,651 individual Raman shift–intensity pairs in one spectrum of a rose leaf), a method which could make use of all these variables would be ideal. Additionally, statistical tests are not appropriate for predicting the group membership of data. For these, we need a different approach—classification algorithms.

### Classification Algorithms

**Figure 3:**
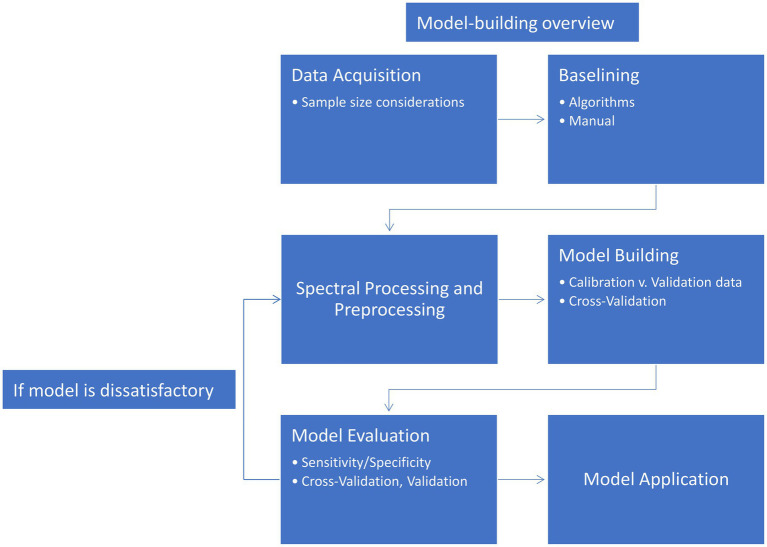
Flowchart of model construction and testing.

A classification algorithm (or classifier) is an organized set of mathematical steps in which the identities of different groups of data are established. These algorithms vary in how this membership is established. At the surface level, classifiers can be divided in two broad categories: unsupervised and supervised. In an unsupervised classification problem, the groupings or classes of samples are not known or otherwise not supplied to the algorithm. This makes the application of these types of models less restrictive, but the results often more difficult to interpret. An unsupervised classifier is ideal for exploring data where relationships are unknown or for providing an initial assessment of relationships in the dataset before further analysis. A supervised classification problem is the exact opposite: The classes of all samples are provided to the algorithm. In a controlled experiment, this is a common scenario. When initially building a classifier, the model first requires data of known origin (control versus infected, for example). Supervised classification is limited by knowledge of the sample, as categories can only be established from experimenter knowledge and may not capture some underlying trends. Additionally, without proper sampling and testing, supervised classifiers can overfit the data, providing overly optimistic classification accuracy. The process of developing a classification model is summarized in Figure 3. The following sections will walk through the figure and elaborate on each cell.

### Sample Size

For multivariate analyses, the performance of the model will typically improve with increasing numbers of spectra in each category (Saccenti and Timmerman, 2016). Additionally, as previously described, co-averaging scans together can improve the signal-to-noise of each individual scan in the sample. However, scanning time greatly limits sample size. We have observed satisfactory performance with approximately 50 scans per sample category, but larger samples are better when possible.

### Baselining

Baseline correction (or “baselining”) involves removal of background signals to improve analysis. It can be thought of as a type of preprocessing step (described in further detail below). Some commercial instruments, such as the Agilent Resolve instrument used by our group, have built-in baselining algorithms. Publicly published algorithms such as automatic weighted least-squares and adaptive iteratively reweighted penalized least squares (airPLS) have shown great results for baseline correction of spectral data (Haaland et al., 1985; Zhang et al., 2010). Baselining can also be done manually in many spectroscopy programs (selecting regions of the spectrum that are baseline by hand). All methods depend on knowledge of where Raman bands are expected to be to prevent data loss or appearance of baseline artifacts as bands. Baselining is important to remove background signals that may influence analysis.

### Preprocessing

Preprocessing steps are treatments applied to the dataset before analysis to ultimately improve classification performance. In this section, we will use a single multivariate classification method (PLS-DA) to evaluate a wide variety of preprocessing steps. These models will be built using the default parameters described in further detail in the PLS-DA section. For the purposes of this analysis, the number of latent variables (LV) will be fixed at 7. Models with 1, 3, or 5 LVs were also tested, but these all performed much worse regardless of the preprocessing steps uses. To facilitate testing, we will use PLS_Toolbox’s Model Optimizer feature, which allows the user to quickly build and compare the results of many different models for the same dataset.

For this analysis, we will evaluate 6 different preprocessing steps as well as a handful of their combinations. These steps are briefly described in Table 1. This is not an exhaustive analysis, but these methods are some of the most commonly used (Verboven et al., 2012). Besides finding the best combination to maximize the model performance, preprocessing steps can help reduce spectral noise, remove acquisition artifacts such as cosmic rays, background from fluorescence. Normalization, for example, is useful for rescaling the data so that overall differences in intensity do not dominate how the model differentiates the data. This is helpful for determining what bands (and therefore molecules) change between each group when classifying the data (Song et al., 2021).

**Table 1:** Description of preprocessing steps tested.

Preprocessing steps	Description
None	No preprocessing beyond automatic baselining by instrument software.
SNV	Standard normal variate scaling—find mean intensity in each spectrum then subtract from all wavenumbers then divide by standard deviation. After scaling, each spectrum will have intensities with mean = 0 and standard deviation = 1.
Mean Center	Find the mean intensity at each wavenumber for the entire dataset and subtract from all spectra. Each wavenumber will have a mean of 0 after this process.
Median Center	Like Mean center but with median instead. Resilient to outliers.
Normalize (Area)	Scale all spectra so the area under the curve of each is 1.
Normalize (Max = 1)	Scale all spectra so the maximum intensity in each is 1.
Derivative	Takes the derivative of each spectrum. Can help reveal overlapping bands and act as baseline correction.

The results of this analysis and brief descriptions of each preprocessing step are presented in Table 2. The performance of each model is evaluated using its F1 score, which indicates better performance as it approaches positive 1. We can see, for the given dataset and model method (PLS-DA), taking the first derivative then mean centering (Analysis 10, bold) resulted in the best performance. The difference between the best and the worst set of steps was 0.13, between normalization (Max =1) and the 1st Derivative + Mean center. We found that 5 methods performed worse than using no preprocessing at all—SNV, median center, both normalization methods alone, and area norm + mean center.

**Table 2:** Performance of all preprocessing combinations tested.

Analysis no.	Preprocessing steps	Average F1 score of cross-validation
1	None	0.8069
2	SNV	0.7582
3	Mean Center	0.8155
4	Median Center	0.7942
5	Normalize (Area)	0.7699
6	Normalize (Max = 1)	0.7593
7	1st Derivative	0.8699
8	SNV, Mean Center	0.8069
9	*Norm (Area), Mean Center*	*0.7804*
10	**1st Der, Mean Center**	**0.8878**
11	1st Der, Median Center	0.8536
12	2nd Der, Mean Center	0.8669
13	Area norm, 1st Der	0.8405

*Bold, Best combination; Italics, combination used in “Applied Examples” section*.

### Model Building

Model building varies from method to method but will generally involve an initial partition of the dataset into “training” and “validation” sets. This partition can be done algorithmically, such as by the Kennard–Stone method, or by simple random selection of data to include in one category or the other (Kennard and Stone, 1969). A model is initially developed using the training dataset, during which the different components of the model (varying method by method) can change with each correct or incorrect classification. This step is then followed by cross-validation, a process by which the training dataset split into smaller training and validation sets to find which version of the model has the best performance. Many cross-validation methods exist, exhibiting different performance levels depending on the model type (Arlot and Celisse, 2010). Following cross-validation, the model is complete and can be applied to the validation data to determine how the model will perform with never-before-seen data.

### Model Evaluation

The performance of a classifying model is typically evaluated by its sensitivity and specificity (Loong, 2003). The sensitivity is the ability of the model to correctly assign data to its known group versus the wrong group. Specificity is the ability of the model to correctly exclude data from groups it is known not belong in versus assigning it to those groups. These values are also known as the true positive rate (TPR) and true negative rate (TNR), respectively. Accuracy can summarize the performance of a model for a given group:


$$Accuracy=\frac{TPR+TNR}{TPR+TNR+FPR+FNR}=\frac{TPR+TNR}{2}$$

where the FPR and FNR are the false positive and negative rates, respectively. The FPR reflects the proportion of samples from a different group assigned to the group in question, while the FNR is the proportion of samples from the group in question incorrectly assigned to a different group. In the previous Preprocessing section, the performance of the model was summarized using the F1 score, which for a given group is defined as:


$$F1=\frac{2TPR}{2TPR+FPR+FNR}$$

For the Applied Examples section, models will be evaluated based on their accuracy for each group. In part 2, they will be evaluated based on the true positive rate instead.

Many other metrics for evaluating models exist. For example, when a model is binary, or contains only two groups, it can be evaluated using the Matthews Correlation Coefficient (MCC), a value that ranges from 1, where all data are assigned correctly, to −1, where all data are assigned to the wrong category. The MCC is defined as:


$$MCC=\frac{TP\times TN-FP\times FN}{\sqrt{\left(TP+FP\right)\left(TP+FN\right)\left(TN+FP\right)\left(TN+FN\right)}}$$

Chicco and colleagues found that the MCC consistently provided the most reliable interpretation of the model compared to either accuracy or MCC (Chicco and Jurman, 2020). This measure is typically limited to binary models but multiclass extensions have been described (Gorodkin, 2004).

These evaluation parameters exist for the calibration, cross-validation, and validation of a model, but either cross-validation or (ideally) validation are reported. If the model performs to the experimenter’s expectations, it can be retained and applied to new data. Otherwise, the preprocessing and model method selection should be revisited to find a model/preprocessing combination with higher classification performance.

### Applied Examples—The Rose Data

This section will describe several types of classification algorithms typically employed with Raman spectral data, then provide examples of their application using the rose dataset described previously. The details of the algorithms are cited with the respective algorithms. All spectra were preprocessed using area normalization and mean centering. For models that have a validation step, cross-validation accuracy with the “most probable” class assignment threshold are reported here. The accuracies of these models per class are summarized in Table 3.

**Table 3:** Performance of all classification models tested on the rose data.

Analysis no.	Method	Settings	Accuracy	SY	HL (Control)	AS	Avg
1	Cluster – Ward’s Method		n/a	n/a	n/a	n/a
2	SVM-C	Cost = 100; Gamma = 3.1623	0.908275	0.860005	0.91939	0.89589
3	SVM-C	Cost = 5e10; Gamma = 3.1263e-4	0.911805	0.86669	0.924645	0.901047
4	KNN	*k* = 3	0.75646	0.6522	0.765585	0.724748
5	KNN	*k* = 5	0.77699	0.67549	0.76551	0.73933
6	KNN	*k* = 12	0.763625	0.70393	0.728525	0.732027
7	KNN	*k* = 3	0.708195	0.663415	0.730175	0.700595
8	KNN	*k* = 4	0.701885	0.68899	0.743955	0.71161
9	KNN	*k* = 10	0.688575	0.72838	0.77635	0.731102
10	SIMCA	AS, HL, SY	0.68253	0.52277	0.696485	0.633928
11	SIMCA	AS, SY, HL	0.680005	0.50373	0.696485	0.62674
12	SIMCA	SY, HL, AS	0.57649	0.50373	0.676385	0.585535
13	SIMCA	SY, AS, HL	0.57649	0.676385	0.50373	0.585535
14	SIMCA	HL, AS, SY	0.68253	0.755105	0.6579	0.698512
15	SIMCA	HL, SY, AS	0.76012	0.755105	0.676385	0.730537
16	SIMCA	Classification Rule – Combined	0.690965	0.70838	0.66145	0.686932
17	SIMCA	Classification Rule – T2	0.596759	0.663845	0.61075	0.623785
18	SIMCA	Classification Rule – Q	0.765035	0.763725	0.666705	0.731822
19	SIMCA	*q* = 0.9; *t* = 0.95	0.78141	0.764245	0.68164	0.742432
20	SIMCA	*q* = 0.85; *t* = 0.95	0.76857	0.752155	0.687765	0.736163
21	SIMCA	*q* = 0.8; *t* = 0.95	0.777235	0.74689	0.690818	0.738314
22	SIMCA	*q* = 0.95; *t* = 0.9	0.769445	0.76398	0.683415	0.738947
23	SIMCA	*q* = 0.95; *t* = 0.85	0.687031	0.73645	0.69922	0.707567
24	SIMCA	*q* = 0.95; *t* = 0.8	0.75961	0.73774	0.707165	0.734838
25	PLS-DA	SIMPLS, no orth, no prior, no weights; 95% confidence	0.859395	0.814955	0.7449	0.806417
26	PLS-DA	model 25 with NIPALs	0.86028	0.816885	0.75628	0.811148
27	PLS-DA	model 25 with DSPLS	0.86028	0.816885	0.75628	0.811148
28	PLS-DA	RobustPLS 0.75	0.82432	0.739675	0.759945	0.774647
29	PLS-DA	RobustPLS 0.75	0.805645	0.75721	0.749905	0.77092
30	PLS-DA	model 25 with hist weights	0.863935	0.828595	0.80203	0.83152
31	PLS-DA	NIPALS hist	0.82287	0.793585	0.78549	0.800648
32	PLS-DA	DSPLS hist	0.82287	0.793585	0.78549	0.800648

### Clustering—Ward’s Method

Clustering is a category of unsupervised classification methods in which data are assembled into groups based on their “distance” from each other. Data typically start in their own clusters which are then linked together by determining which clusters are the closest to each other. This process is repeated until all data are united in one large cluster containing many smaller clusters. In Ward’s method, clusters are formed by determining which new clusters would result in the lowest within-cluster variance (Ward, 1963).

**Figure 4:**
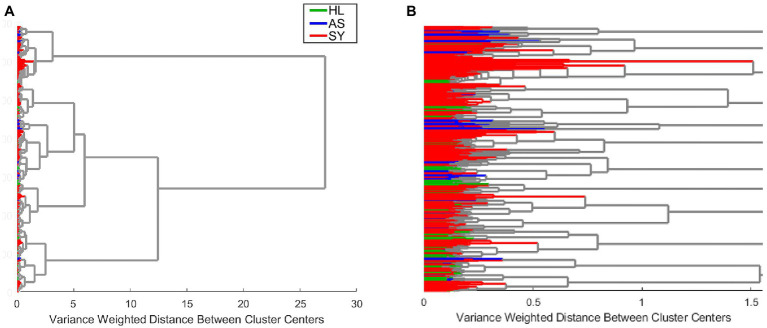
Example clustering dendrogram at full size **(A)** or zoomed in to reveal the details of individual clusters **(B)**. HL—healthy control; AS—asymptomatic; SY—symptomatic.

**Figure 5:**
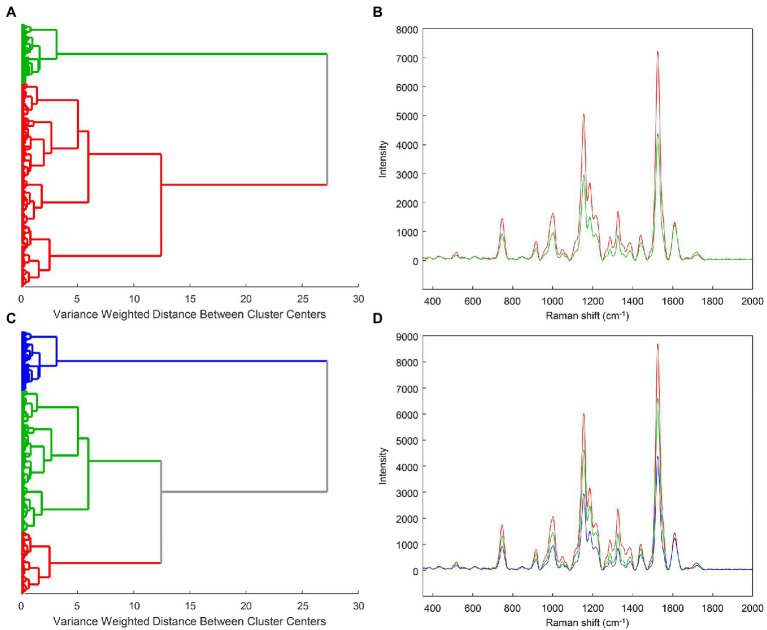
Example cluster dendrograms and spectra for 2 **(A,B)** or 3 **(C,D)** clusters.

We used clustering by Ward’s method to analyze the entire example rose dataset. As indicated by the overall cluster dendrogram (Figure 4), no class-specific trends are captured by the clustering. This is further confirmed by evaluating the spectra of different proposed clusters. Whether using two or three clusters (Figure 5), the spectral differences across groups are primarily associated with variations in intensity. Additionally, the lack of variation at the 1,720 cm^−1^ provides further evidence that the observed clusters may not be related with the known categories in the data. These cluster results indicate that the spectra cluster with each other based on their intensities, rather than their known categories.

**Figure 6:**
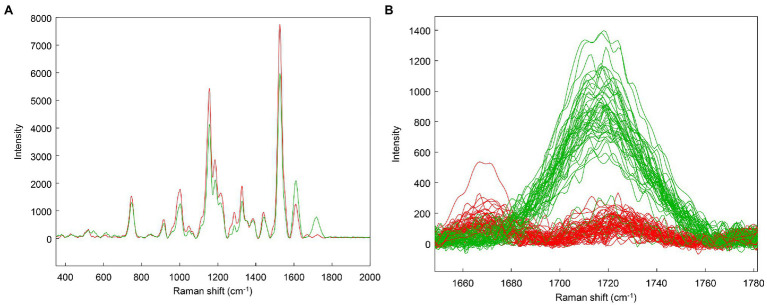
Clustering on the asymptomatic (AS) class only. Spectral averages **(A)** and zoom of the 1720 cm^−1^ region with individual spectra **(B)**.

However, when clustering only the asymptomatic data, an interesting trend is revealed: One cluster has a stronger 1720 cm^−1^ band than the other (Figure 6). This suggests that the asymptomatic class may represent different stages of infection: Those having weaker 1,720 cm^−1^ bands perhaps may be earlier in infection, while those plants with increased intensity at 1,720 cm^−1^ may have more advanced infections. For the purposes of the subsequent analyses, these two groups will be maintained as a single category as they were when originally analyzed (Farber et al., 2019a). Clustering helped to identify an interesting trend in the data that the original label did not capture. The main advantage of clustering is to quickly identify trends in datasets that are not immediately apparent, particularly when group membership is unknown.

#### Support Vector Machine Classification

Support vector machines (SVM) are a family of classification algorithms initially designed for binary classification. In brief, they function by finding the hyperplane (roughly, a line in multidimensional space) that separates the categories the best. This hyperplane is selected to maximize its distance from the datapoints in each category to improve its ability to predict never-before-seen data. This hyperplane also has a “soft margin,” which allows samples to appear near, but on the incorrect side of, the hyperplane without greatly influencing the results. Extensions of SVM enable the algorithm to handle classification of more than two categories (Noble, 2006).

**Figure 7:**
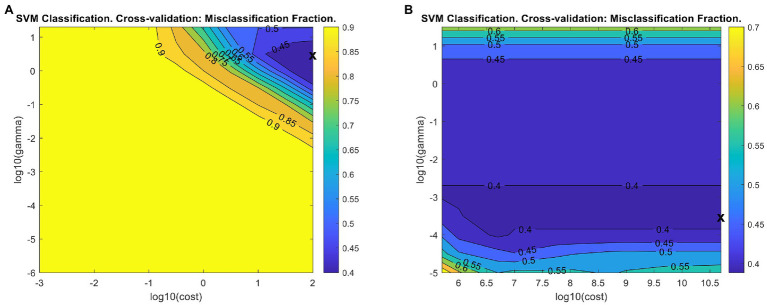
Misclassification fraction plots for the initial **(A)** and final **(B)** support vector machine classification (SVM-C) models. X - The gamma/cost pair reported in the final model.

We conducted support vector machine classification (SVM-C) using LIBSVM implemented in PLS_Toolbox (Chang and Lin, 2011). SVM-C requires initial optimization of two parameters: C (cost), a value associated with the soft margin, and gamma, associated with the shape of the hyperplane. In PLS_Toolbox, the model is initially provided with 9 cost and gamma values to iterate through which can be customized by the user. To facilitate rapid parameter optimization, we reduced the number of cross-validation iterations to make the model building process faster. After each iteration of SVM-C in PLS_Toolbox, a misclassification fraction plot (Figure 7A) is generated, illustrating the misclassification percent from each gamma-cost pair. The X on the plot indicates the gamma-cost pair used in the final model. The plot can be used to determine whether the final gamma-cost pair was the most optimal. Ideally, the X should be toward the middle of the plot, rather than on an edge. We increased the maximum cost value tested to shift the plotted region to the right of the initial region, but the model failed to build due to hardware limitations when excessively large cost values were used. The final optimized model misclassification plot indicates further optimization is possible, but such optimization would require better computational resources (Figure 7B). The final model showed slight accuracy increases for all three classes compared to the initial model (Table 4).

**Table 4:** Performance of the best classification model of each type on the Rose data.

Analysis no.	Method	Accuracy			
SY	HL (Control)	AS	Avg
3	SVM-C	0.911805	0.86669	0.924645	0.901047
30	PLS-DA	0.863935	0.828595	0.80203	0.83152
19	SIMCA	0.78141	0.764245	0.68164	0.742432
9	KNN	0.688575	0.72838	0.77635	0.731102
P-A	PLS-DA	0.913235	0.913235	0.913235	0.913235
P-B	PLS-DA	n/a	0.92172	0.92217	0.921945
P-aggregate					0.91759

*P-A and B—Previous models A and B originally described in Farber et al. (2019a). P-aggregate—averaged accuracy of P-A and P-B averages*.

#### *k*-Nearest Neighbors

*k*-nearest neighbors (KNN) classification is a method in which class assignment is determined by the class label of the *k* nearest neighbor points in space (Kramer, 2013). The model is initially trained to develop a set of patterns to compare new samples to. After training, new samples are compared to their nearest *k* neighbors to determine class membership (Laaksonen and Oja, 1996). For example, if *k* = 3, then datapoint X is assigned to a class based on the three nearest neighbors to it. This method can also be used for clustering and regression.

**Figure 8:**
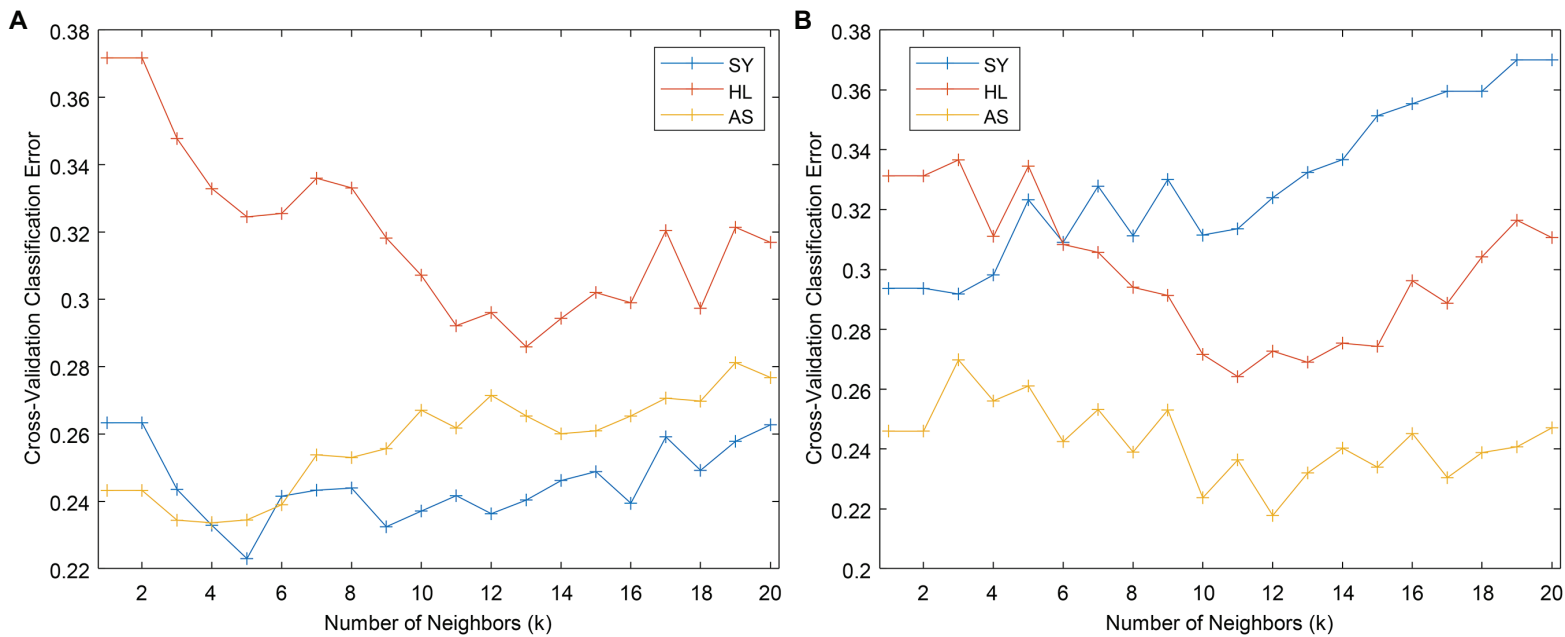
Cross-validation error versus *k* plots for the full **(A)** and even **(B)** datasets.

We used the KNN classifier implemented in PLS_Toolbox to conduct KNN classification. One user-defined parameter, *k*, can be optimized to improve model performance. We used the default value of *k* = 3 to obtain the *k* versus cross-validation (CV) error plot (Figure 8A). This plot shows how the error rate of CV changes with the number of neighbors selected. We then selected two additional *k* values to test: 5 and 12. 5 was selected because it shows local minima in all three curves, while 12 is near the minimum for HL, which has the largest error of the three groups. From Table 4, *k* = 5 is an improvement over 3, but 12 demonstrates tradeoffs in accuracy—while SY and AS accuracy goes down, HL accuracy increases (as previously indicated by Figure 8A). Despite these changes in accuracy, changing the *k* value does not dramatically alter the overall model accuracy.

*k*-nearest neighbors classification can suffer when one class contains many more samples than the others. In the current dataset, the SY class is twice as large as the other two classes put together. To attempt to combat any effects this might have on the classification, we reduced the SY class to 120 samples using the Kennard–Stone algorithm (Kennard and Stone, 1969). Using the even dataset, We built a new KNN model using *k* = 3 to obtain the *k* versus CV error plot (Figure 8B). Based on the plot, we decided to build models using *k* = 4 and 10, where local minima in error were observed. These models showed slight improvement for classification of the HL and AS but had markedly worse performance in classifying the SY category (Table 4).

#### Soft Independent Modeling of Class Analogy

Soft independent modeling of class analogy (SIMCA) is a classification method built on principal component analysis (PCA; Wold and Sjöström, 1977). PCA is a method in which the most important parts of the data are identified and retained to compress and simplify the dataset. These important parts of the data are called principal components (PCs; Abdi and Williams, 2010) In SIMCA, a PCA model is built for each class (or combination of classes) to identify the PCs. These PCs are then used in the SIMCA classification.

We attempted to optimize a SIMCA model for the rose data. PCA and SIMCA analyses were conducted using the methods as implemented in PLS_Toolbox. The first step in building the SIMCA model is to assemble the initial PCA models for each class. For this example, the number of PCs in each model was selected that approximately 90% of the variance was explained. This was 4, 5 and 5 PCs for AS, HL, and SY, respectively. After assembling the three PCA models, we then built the SIMCA model. For all SIMCA models, both the Hotelling T^2^ value and Q residuals were used in the decision rule for classification. The confidence limits for both values were set at 95%. We found that the performance of the model varied dramatically with the order that the three classes were modeled. The model assembled using the order HL, SY, AS showed the best overall performance of the SIMCA models, but no model performed exceptionally.

Using the HL, SY, and AS order, we further optimized the SIMCA model. First, the type of decision rule was tested. Decision rules are how a model determines which data belongs in a class. For SIMCA in PLS_Toolbox, the decision rule determines which values are used as distance measures. Options include: Hotelling T^2^ values, Q residual values, both T^2^ and Q (both), or a combined measure derived from T^2^ and Q (combined). The initial model used both values, so the other three options were tested. We found that both and Q only performed similarly, while the combined and T^2^ options performed worse. Next, using “both” as the decision rule, we varied the confidence limit for T^2^ and Q from their initial values of 0.95 to find the optimal combination. We found that reducing the Q value to 90% while leaving T at 95% slightly improved the model (Table 4).

#### Partial Least Squares Discriminant Analysis

Partial least squares discriminant analysis (PLS-DA) is a frequently used classification method for spectroscopic data (Shashilov and Lednev, 2010). It combines linear discriminant analysis (LDA) with the multicollinearity tolerance of partial least squares. LDA is a robust method that works by maximizing the ratio of between-class variance to within-class variance to guarantee maximum separation between groups (Izenman, 2008). It is normally limited by datasets with high multicollinearity, or those where many explanatory variables have linear relationships with each other. Raman data often highly multicollinear—the intensities of Raman shifts near each other (1,525 and 1,527 cm^−1^, for example) tend to have linear relationships. PLS-DA works well with multicollinear data, making it well-suited for Raman data analysis (Lee et al., 2018).

We reanalyzed this rose dataset using PLS-DA. While it has been previously analyzed using this method, in this case, we will maintain three separate groups instead of combining groups to improve accuracy (Farber et al., 2019a). In PLS_Toolbox, the user-defined parameters for PLS-DA include: which PLS algorithm to use; whether the model is orthogonalized or not; and sample weighting. We calibrated the initial model using the default parameters for PLS-DA: SIMPLS algorithm, no orthogonalization, and no weights. We found that SIMPLS with weighted classes performed the best, though the difference was not large. While orthogonalization is generally an improvement over regular PLS-DA, it does not impact the overall accuracy of the model (Bylesjö et al., 2006).

**Figure 9:**
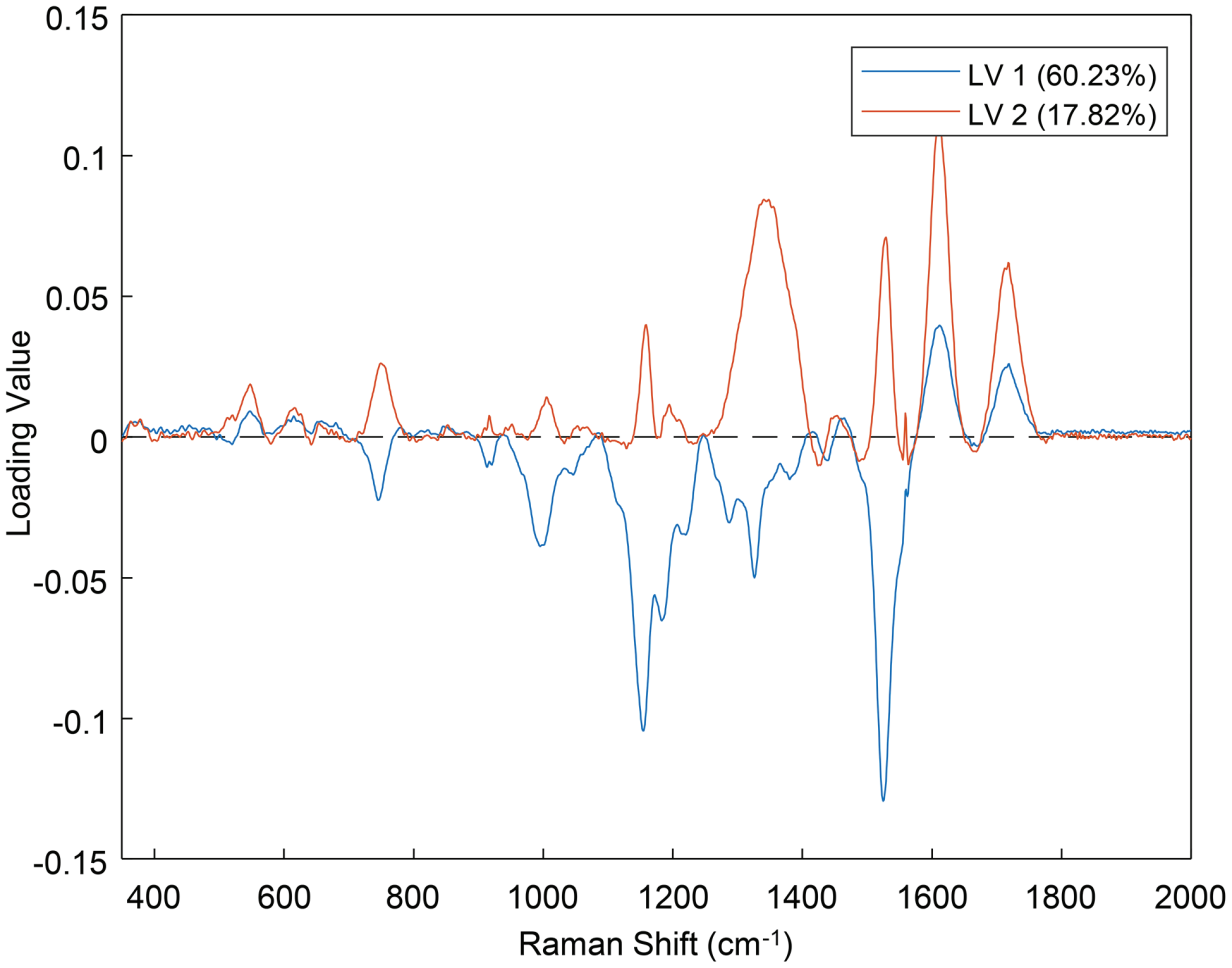
Loadings plot for the first two latent variables (LVs) of the best performing PLS-DA model.

One of the major outcomes of PLS-DA is the loadings plot, which indicates which parts of the data were the most important for the prediction (Eriksson et al., 2013). Each latent variable (LV, analogous to a principal component) of the model has its own loadings plot (Figure 9). The greater the absolute distance of a point from the zero-line, the more important that position was for the prediction. For example, in Figure 9, the band near 1,525 cm^−1^ was important in both LVs 1 and 2, but the region around 1,350 cm^−1^ was more important for LV 2 than LV 1. Because the loadings plots are basically a type of Raman spectra, they can be used to determine what spectral regions might change from class to class.

## Conclusion

The results of the best models from each method tested (excluding clustering) are summarized in Table 4. With the preprocessing steps selected, SVM-C was able to achieve 90% classification accuracy across the three classes. However, every modelling method, sample set, and preprocessing step set are different. Different sets of preprocessing steps may have improved the performance of the other modeling methods. Alternative approaches to applying the data may also improve the outcome.

For example, in the publication for which the rose data were generated, a pair of PLS-DA models was used for the overall classification (P-A and P-B in Table 4). The first model (SA) discriminated between HL + AS and SY, while the second (HA) separated HL and AS. Each step also employed different preprocessing steps to maximize the accuracy of classification. These two models together performed similarly to the single SVM-C model (though the SVM was limited by available hardware). Ultimately, we found that without combining groups, SVM-C was the best method to classify these data. SVM-C should not be regarded as the best method for everything, however. Instead, many different methods should be tested before finally deciding which to use. We also suggest performing cross-validation of models to avoid the overfitting. The alternative approach is data partitioning into training and validation sets, where the training set is used for model development and the validation set is used for external validation (Payne et al., 2021). From the perspective of disease sensing, the presented results describe a simple scenario of rose rosette diagnostics that did not take into consideration other factors, such as geographical location of plants, plant variety abiotic stresses that plants could face. We infer that consideration of these factors is required to develop Raman spectroscopy into robust and reliable sensing approach. Finally, the authors want to note that all raw data for the reported above analyses can be available upon request.

## Data Availability Statement

The raw data supporting the conclusions of this article will be made available by the authors, without undue reservation.

## Author Contributions

CF performed data collection, data analysis, and manuscript writing. DK performed manuscript writing and supervision. All authors contributed to the article and approved the submitted version.

## Funding

This work was supported by the Governor’s University Research Initiative (GURI) grant program of Texas A&M University, GURI Grant Agreement No. 12-2016, M1700437.

## Conflict of Interest

The authors declare that the research was conducted in the absence of any commercial or financial relationships that could be construed as a potential conflict of interest.

## Publisher’s Note

All claims expressed in this article are solely those of the authors and do not necessarily represent those of their affiliated organizations, or those of the publisher, the editors and the reviewers. Any product that may be evaluated in this article, or claim that may be made by its manufacturer, is not guaranteed or endorsed by the publisher.
